# Synthesis and Characterization of Naphthalenediimide-Functionalized Flavin Derivatives

**DOI:** 10.3390/ijms14047468

**Published:** 2013-04-03

**Authors:** Nada Zainalabdeen, Brian Fitzpatrick, Mohanad Mousa Kareem, Vikas Nandwana, Graeme Cooke, Vincent M. Rotello

**Affiliations:** 1 Glasgow Centre for Physical Organic Chemistry, WestCHEM, School of Chemistry, University of Glasgow, Glasgow G12 8QQ, UK; E-Mails: n_y92@yahoo.com (N.Z.); brianf@chem.gla.ac.uk (B.F.); mohanad_1972@yahoo.com (M.M.K.); 2 Department of Chemistry, University of Massachusetts, Amherst, MA 01003, USA; E-Mail: nandwana@chem.umass.edu

**Keywords:** flavin, naphthalenediimide, redox, optical

## Abstract

Two acceptor–acceptor dyads have been synthesized featuring a flavin moiety and a naphthalenediimide (NDI) unit. The NDI unit is linked to the flavin through a short spacer group via either the N(3) or N(10) positions of the flavin. We have investigated the UV-Vis and redox properties of these multi-electron accepting systems which indicate that these materials display the collective properties of their component systems. Fluorescence spectroscopy measurements have revealed that their emission properties are dominated by the flavin unit.

## 1. Introduction

Flavoenzymes are an important class of redox-active enzymes that are responsible for maintaining a range of cellular processes [1]. For example, they have been shown to play a significant role in the dehydrogenation, hydroxylation and oxidation of a range of biological substrates and one and two-electron transfer reactions both to and from other redox centers [2]. In the majority of systems, the main flavin co-factor present is either flavin adenine dinucleotide (FAD) or flavin mononucleotide (FMN), which is usually non-covalently bound to the active site of the enzyme through an array of specific non-covalent interactions. In addition to providing a scaffold to facilitate catalysis and electron transfer reactions, the protein backbone of the enzyme has been shown to tune the redox [3] and fluorescence properties of the flavin unit [4]. A variety of biomimetic model systems have been developed to determine the specific role non-covalent interactions play in modulating these processes [5,6].

**Figure 1:**
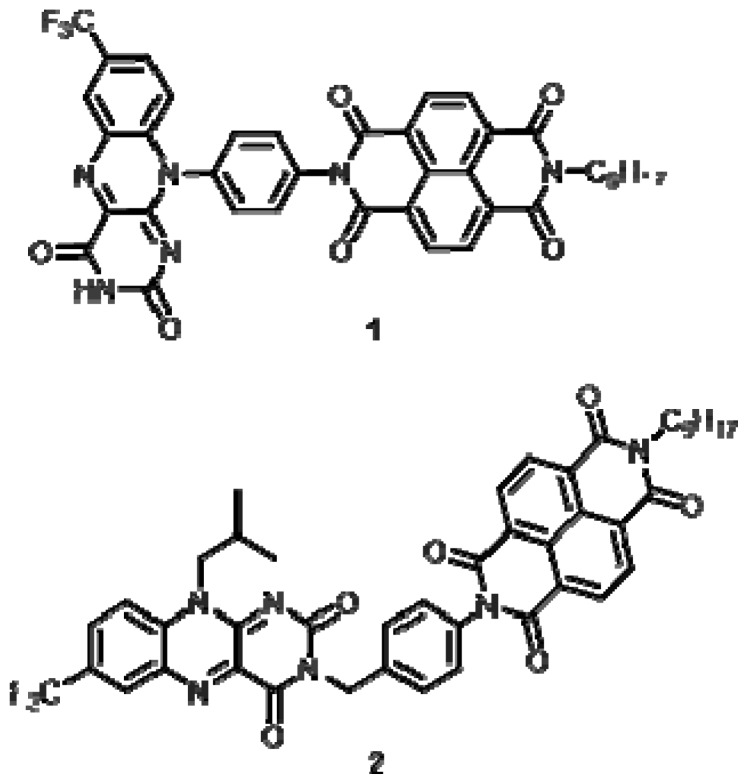
Structures of compounds **1** and **2**.

The optical and electronic properties of flavins make them excellent starting points for the design of functional molecular systems. A number of examples of flavin derivatives that are covalently linked to other acceptor systems have been reported that feature interesting and useful optical and electrochemical properties [7,8]. Herein we report the synthesis and characterization of two acceptor-acceptor systems that feature covalently linked naphthalenediimide (NDI) [9,10] and flavin moieties [11]. In these compounds, the NDI unit is linked to the flavin through a short aromatic spacer group and is connected to the flavin either at the N(10) or N(3) positions to afford compounds **1** and **2**, respectively. While the structures of these two materials are related, these two compounds let us compare conjugated (**1**) and non-conjugated (**2**) flavin-NDI constructs (Figure 1).

## 2. Results and Discussion

### 2.1. Synthesis

**Scheme 1:**
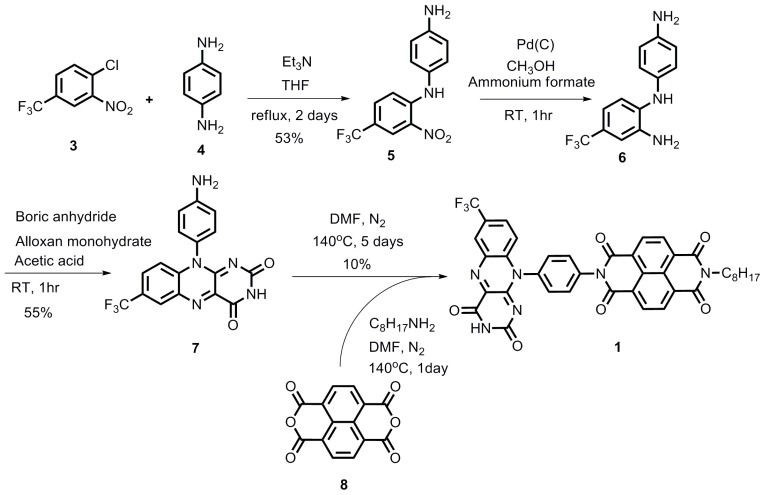
Synthesis of compound **1**.

**Scheme 2:**
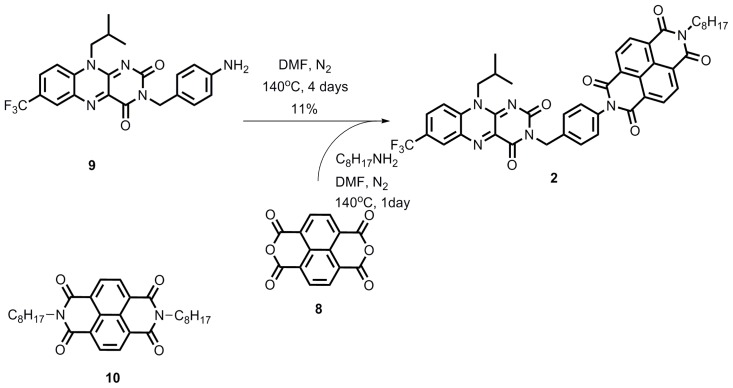
Synthesis of compound **2**.

The synthesis of the flavin derivatives **1** and **2** is provided in Schemes 1 and 2, respectively. Compound **5** was synthesized in acceptable yield from commercially available **3** and **4**. Reduction of **5** using ammonium formate afforded compound **6**, which was subsequently converted without further purification to flavin derivative **7** in moderate yield. This flavin together with octylamine was reacted with dianhydride **8** in a one-pot procedure to furnish compound **1**[12]. Compound **2** was prepared similarly using flavin **9**[13], octylamine and compound **8**.

### 2.2. Characterization

**Figure 2:**
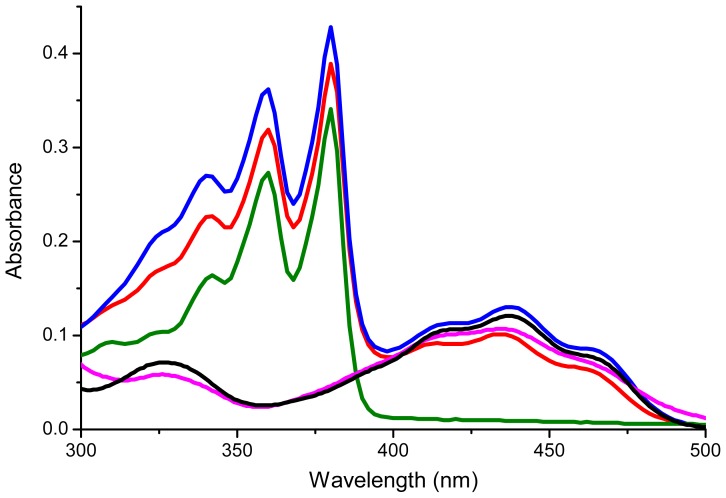
UV-Vis spectra of compound **1** (red line), **2** (blue line), **10** (green line), **7** (magenta line) and **9** (black line). Recorded in DCM at ~1 × 10^−5^ M.

UV-Vis spectra of **1**, **2**, **7**, **9** and **10** were recorded in CH_2_Cl_2_ (~1 × 10^−5^ M) (Figure 2), and the key absorptions are provided in Table 1. Compounds **1** and **2** displayed two strong absorption peaks at around 360 nm and 380 nm that are characteristic of the NDI unit [14]. In addition, absorption peaks were observed at around 414, 434 and 455 nm for compound **1** and 420, 437 and 462 nm for compound **2** which can be attributed to the flavin moiety [15–17]. Thus the UV-Vis data indicate that linking the flavin and NDI units together in this way provide systems covering a wider swath of the spectrum compared to the individual parent flavin and NDI units.

**Figure 3:**
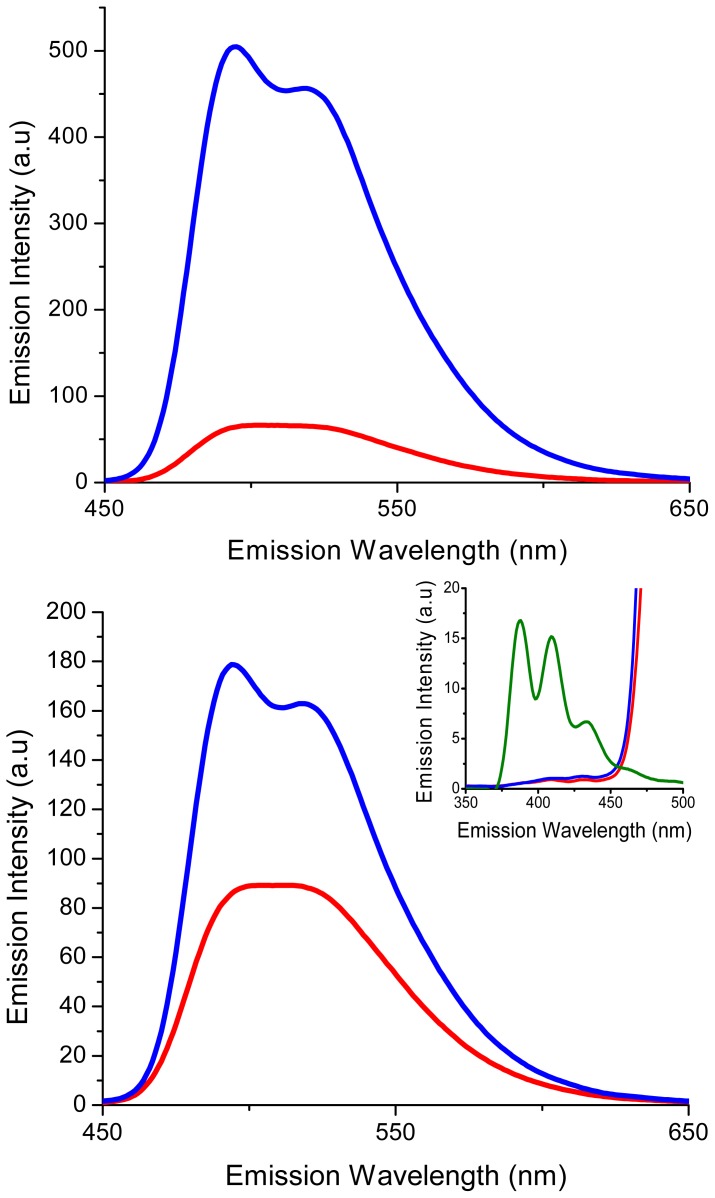
Fluorescence emission spectra of compounds **1** (red line) and **2** (blue line). Recorded at excitation wavelengths: λ_ex_ = 435 nm (top) λ_ex_ = 380 nm (bottom). Recorded in CH_2_Cl_2_ (1 × 10^−5^ M). Inset shows the emission spectrum of **1**, **2** and **10** (green line) between 375 and 450 nm (excited at 380 nm).

The fluorescence emission spectra of compounds **1** and **2** were recorded upon excitation at 435 nm (excitation of flavin unit) and 380 nm (excitation of NDI and flavin units) (Figure 3) [18]. In both cases, the intensity of the emission between 450 and 650 nm was greater for compound **2** compared to **1**, presumably due to intramolecular quenching of the flavin fluorophore. As expected, when **1** and **2** were excited at 435 nm, emission occurred around 450–650 nm which is typical for flavin units. However, when excited at 380 nm, limited emission for the NDI moiety (Figure 3 inset) between 375–450 nm was observed, with the fluorescence properties of the flavin unit dominating the emission properties of these systems.

**Figure 4:**
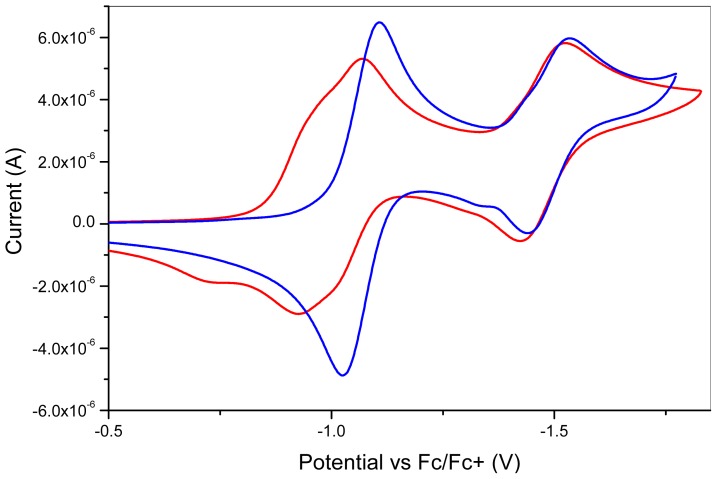
Cyclic voltammetry of compounds **1** (red line) and **2** (blue line).

Electrochemical properties of **1** and **2** were investigated using cyclic voltammetry (CV) (Figure 4) and were compared to that of NDI **10** and to the parent flavin units **7** and **9** (Table 2). Compound **1** displayed two closely overlapping pseudoreversible waves at *E*_½_ = −0.95 V and −0.99 V corresponding to the formation of the flavin [19] and NDI radical anions, and a single one-electron redox wave at −1.47 V that presumably corresponds to the formation of the NDI dianion [14]. Compound **2** on the other hand displayed two pseudoreversible redox waves *E*_½_ = −1.07 V and −1.53 V, with the first two-electron redox wave corresponding to the near-simultaneous formation of the flavin and NDI radical anion states, while the second redox wave was observed at a more negative potential due to the formation of the NDI dianion. When compared to the redox waves obtained for the parent flavins (**7** and **9**) and NDI (**10**), only small changes in half-wave potentials were observed. For example, compound **1** displayed around a 50 mV lower reduction potential for the NDI dianion state compared to **10**, which is presumably a consequence of the electron withdrawing nature of the flavin unit when linked to the NDI unit in this way [20].

**Figure 5:**
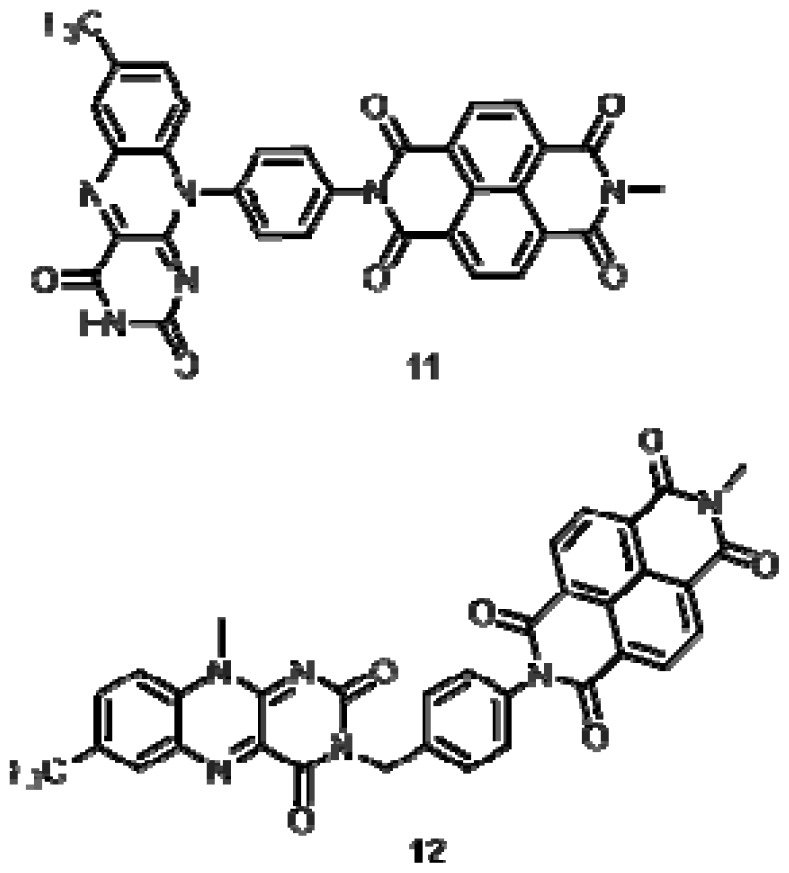
Structures of compounds **11** and **12**.

**Figure 6:**
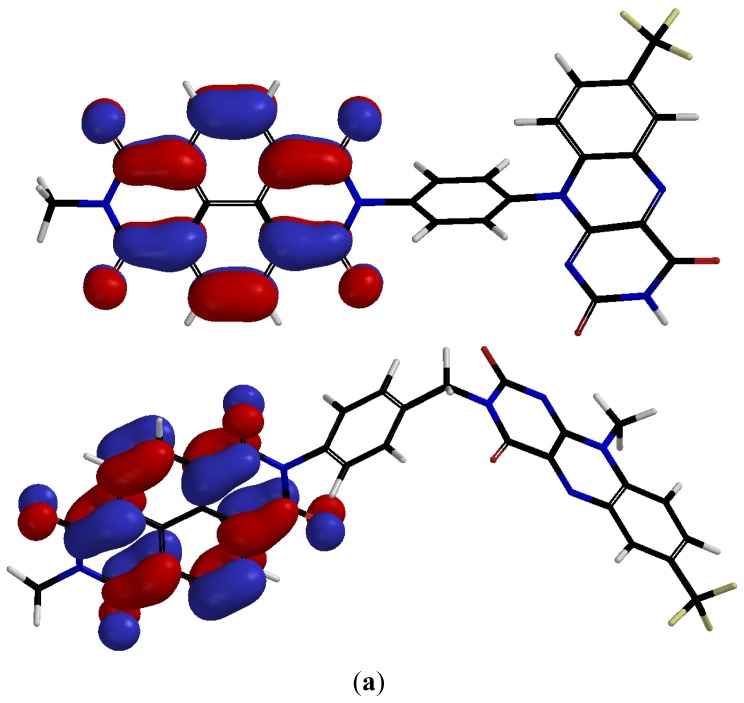

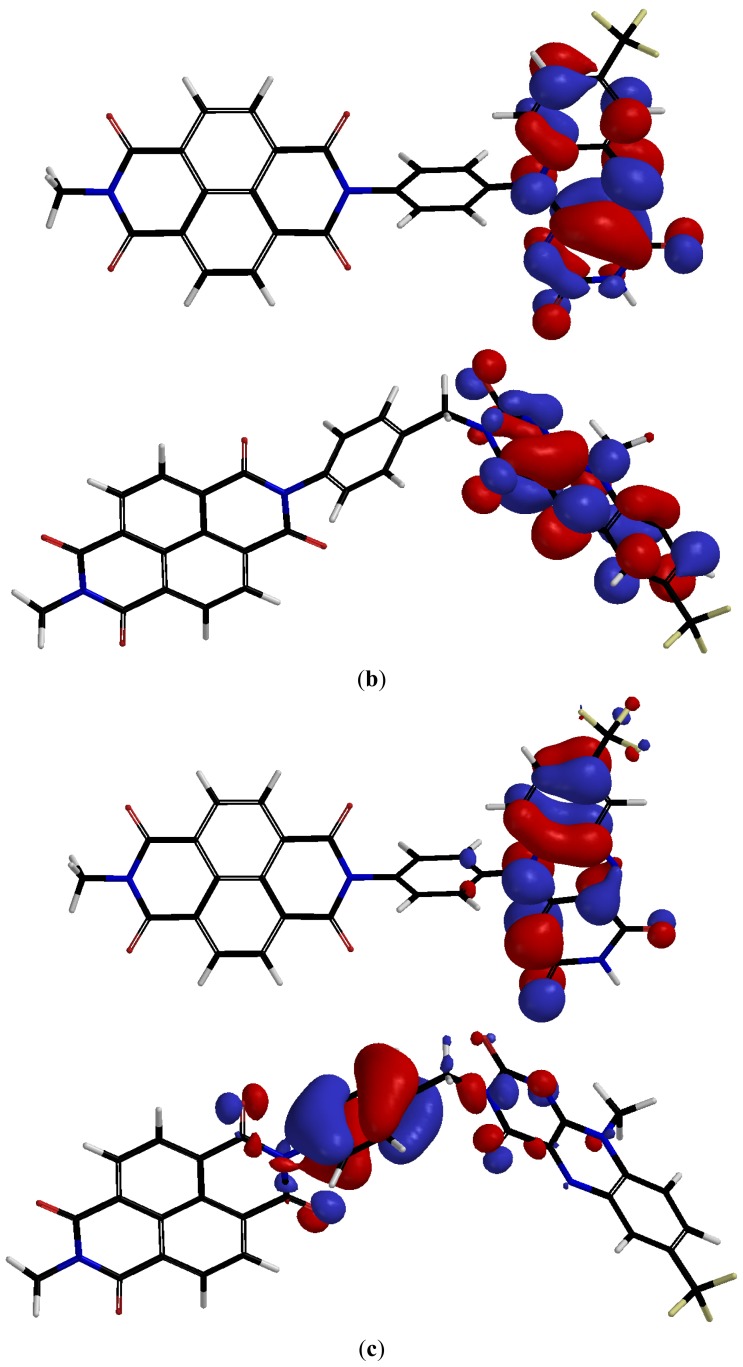
B3LYP predicted (**a**) LUMO; (**b**) LUMO +1 and (**c**) HOMO maps of compounds **11** and **12**.

We have modeled the structure of model compounds **11** and **12** (Figure 5) to probe the electronic properties of the parent dyads **1** and **2**. The LUMO, LUMO + 1 and HOMO maps for both compounds are presented in Figure 6. In both cases, the models suggest that the LUMO is located over the NDI unit of the dyad, consistent with the observation that this unit dominates the electron-accepting properties of these systems. The LUMO + 1 for both derivatives is localized over the flavin moiety. However, the HOMO maps differ significantly for **11** and **12**, with the HOMO largely residing over the flavin nucleus of **11** and over the bridging benzylamine moiety of compound **12**.

## 3. Experimental Section

All reactions were carried out under an inert atmosphere using oven-dried glassware. All solvents for the reactions were of reagent grade. Flash column chromatography was carried out using silica gel as the stationary phase. Melting points are uncorrected. The UV-Vis spectra were recorded using a Perkin Elmer Lambda 25 and the fluorescence measurements were recorded using a Shimadzu RF5301 instrument. The CV data were recorded at room temperature on a CH-Instruments 440 Electrochemical Analyser. CVs were recorded at a concentration of 1 × 10^−4^ M in CH_2_Cl_2_ containing tetra-n-butyl ammonium hexafluorophosphate (Bu_4_NPF_6_ 0.1 M) as supporting electrolyte with a platinum working and counter electrodes and a silver wire as a quasi-reference electrode. The *E*_½_ values for the compounds were determined relative to the ferrocenium/ferrocene (Fc^+^/Fc) redox couple (used as an internal standard). The Fc^+^/Fc couple was adjusted to 0.0 V and the reduction potentials presented in Table 2 are relative to this value. Spartan ‘08 (Wavefunction Inc., Irvine, CA, USA) was used to model the structures of compounds **11** and **12** (analogues of **1** and **2** where the octyl and isobutyl chains have been shortened to a methyl unit to facilitate faster calculation times). The structures were optimized using RB3LYP/6-31G(D) methodology.

### Compound **5**

1-Chloro-2-nitro-4-(trifluoromethyl)benzene (**3**) (5.00 g, 24.27 mmol), *p*-phenylenediamine (**4**) (2.40 g, 22.17 mmol) and triethylamine (3 mL, 22.17 mmol) were dissolved in THF (60 mL) and were heated under reflux for 2 days. The NEt_3_·HCl salt which formed was removed by filtration and the organic solvent was removed under reduced pressure. The residue was dissolved in DCM, washed with water (2 × 100 mL) and dried with MgSO_4_, filtered and the solvent was concentrated under reduced pressure. Purification by column chromatography (silica gel: eluting with petroleum ether/ethyl acetate 7:3) afforded **5** as a red powder (3.50 g, 53%), mp 116–118 °C. ^1^H NMR (400 MHz, CDCl_3_) δ = 9.49 (s, 1H, NH), 8.42 (s, 1H, CH), 7.40 (d, 1H, *J* = 9.2 Hz, CH), 6.98 (d, 2H, *J* = 8.4 Hz, CH), 6.96 (d, 1H, *J* = 8.4 Hz, CH), 6.68 (d, 2H, *J* = 8.4 Hz, CH), 3.73 (s, 2H, NH). ^13^C NMR (100 MHz, CDCl_3_) δ = 146.8, 145.8, 131.6, 127.8, 127.6, 124.7, 116.7, 116.0. MS (FAB/NOBA (M + H)^+^) *m*/*z* 298.4. ν_max_/cm^−1^ 3414*s*, 3349*s*, 3221*w*, 3016*m*, 2970*w*, 1738*w*, 1631*s*, 1573*s*, 1506*s*, 1357*m*, 1321*s* (C-NO_2_), 1226*m*, 1091*m*, 919*s*, 827*m*, 763*s*, 686*s*. Anal. Calc. for C_13_H_10_F_3_N_3_O_2_: C 52.52%, H 3.37%, N 14.14%. Found: C 52.50%, H 3.35%, N 14.08%.

### Compound **6**

Compound **5** (3.50 g, 11.79 mmol), Pd(C) 10% (0.30 g) and ammonium formate (4.50 g, 71.43 mmol) were dissolved in methanol (60 mL) and were left stirring at room temperature for 1 h. The Pd(C) was removed by filtration and the filtrate was concentrated under reduced pressure. The residue was washed with DCM to precipitate the ammonium formate, which was removed by filtration. The organic solution was then concentrated under reduced pressure to produce a red solid (**6**) (3 g). No further purification was undertaken due to the instability of the compound.

### Compound **7**

Compound **6** (3.00 g, 11.24 mmol), boric anhydride (0.78 g, 11.24 mmol) and alloxan monohydrate (1.6 g, 11.20 mmol) were dissolved in glacial acetic acid (50 mL) and the resulting solution was stirred at room temperature for 1 h. The product was collected by adding DCM and then extracted from the reaction mixture. The mixture was dried over magnesium sulfate, filtered and the solvent was removed under reduced pressure. Purification by column chromatography (silica gel: eluting with DCM/petroleum ether 9:1) afforded **7** as a dark red powder (0.24 g, 55%), mp > 300 °C. ^1^H NMR (400 MHz, DMSO) δ = 11.52 (s, 1H, NH), 8.51 (s, 1H, CH), 8.03 (dd, 1H, *J* = 1.6 Hz, 9 Hz, CH), 7.09 (d, 1H, *J* = 8.8 Hz, CH), 7.00 (d, 2H, *J* = 8.4 Hz, CH), 6.78 (d, 2H, *J* = 8.4 Hz, CH), 5.63 (s, 2H, NH_2_). ^13^C NMR (100 MHz, DMSO) δ = 159.3, 155.6, 152.7, 150.1, 141.2, 137.5, 133.8, 129.9, 128.5, 127.9, 125.8, 125.5, 124.9, 123.1, 122.2, 118.7, 114.3. MS (FAB/NOBA (M + H)^+^) *m*/*z* 373.8. ν_max_/cm^−1^ 3470*w*, 3347*s*, 3016*m*, 2970*s*, 2948*w*, 1737*w*, 1653*m*, 1538*m*, 1505*m*, 1446*m*, 1345w, 1293*s*, 1229*w*,1110*s*, 1065*s*, 903*s*, 841*s*, 765*s*. Anal Calc. for C_17_H_10_F_3_N_5_O_2_: C 54.69%, H 2.68%, N 18.76%. Found: C 54.42%, H 2.68%, N 17.67%.

### Compound **1**

A solution of naphthalene dianhydride (**8**) (0.30 g, 1.34 mmol) in dry DMF (50 mL) was heated to 140 °C under N_2_ atmosphere. octylamine (0.19 g, 1.34 mmol) was then added drop wise. The reaction mixture was heated under reflux for 24 h. Compound **7** (0.50 g, 1.34 mmol) was then added. After 5 days heating under reflux, the reaction mixture was cooled and the DMF was removed under reduced pressure. Purification by column chromatography (silica gel: eluting with DCM/acetone 9:1) and subsequent recrystallization from diethyl ether afforded **1** as a yellow/brown powder (0.10 g, 10%), mp 190–192 °C. ^1^H NMR (400 MHz, CDCl_3_) δ = 11.65 (s, 1H, NH), 8.75 (s, 4H, CH_NDI_), 8.61 (s, 1H, CH_ar_), 8.12 (dd, 1H, *J* = 2.1 Hz, 9.2 Hz, CH_ar_), 7.82 (d, 2H, *J* = 8.6 Hz, CH_ar_), 7.63 (d, 2H, *J* = 8.6 Hz, CH_ar_), 6.98 (d, 1H, *J* = 9.2 Hz, CH_ar_), 4.09 (t, 1H, *J* = 7.4 Hz, 9.5 Hz CH_2_). 1.69 (br, 2H, CH_2_), 1.23–135 (br, 10H, CH_2_), 0.87 (t, 3H, *J* = 5.2 Hz, 7Hz, CH_3_). ^13^C NMR (100 MHz, CDCl_3_) δ = 162.8, 162.7, 159.1, 131.3, 130.5, 128.5, 126.7, 126.5, 117.8, 40.2, 31.2, 28.7, 28.6, 27.4, 26.5, 22.1, 13.9. MS [FAB/NOBA (M + H)^+^] *m*/*z*: 735.8. HRMS (FAB/NOBA), C_39_H_29_N_6_O_6_F_3_: Calcd: 735.2179, found. 735.2173. ν_max_/cm^−1^ 3357*w*, 3082*m*, 2923*m*, 2855*w*, 1791*w*, 1716*m*, 1659*s*, 1595*s*, 1549*m*, 1333s, 1305*m*, 1248*m*, 1202*s*, 1166*s*, 1129*m*, 1068*s*, 841*m*, 767*s.*

### Compound **2**

Naphthalene dianhydride (**8**) (0.36 g, 1.35 mmol) was dissolved in dry DMF (30 mL). The solution was heated to about 140 °C under N_2_ atmosphere. To this octylamine (0.18 g, 1.35 mmol) was added drop wise for about 10 min and the reaction mixture was heated under reflux for 24 h. Compound **9** (0.3 g, 0.68 mmol) was added and the mixture was left heating under reflux for another 4 days. The reaction mixture was cooled down and the DMF was removed under reduced pressure. Purification by column chromatography (silica gel: eluting with 100% DCM) followed by recrystallization from diethyl ether afforded **2** as a yellow/brown product (0.06 g, 11%), mp 190–192 °C (dec). ^1^H NMR (400 MHz, CDCl_3_) δ = 8.78 (s, 4H, CH_NDI_), 8.59 (d, 1H, *J* = 1.2 Hz, CH_ar_), 8.04 (dd, 1H, *J* = 2 Hz, 9 Hz, CH_ar_), 7.89 (d, 2H, *J* = 8.4 Hz, CH_ar_), 7.76 (d, 1H, *J* = 8.8 Hz, CH_ar_), 7.28 (d, 2H, *J* = 8.4 Hz, CH_ar_), 4.62 (br, 2H, CH_2_). 4.21 (t, 2H, *J* = 7.6 Hz, 9.3 Hz, CH_2_), 2.48 (m, 1H, CH), 1.76 (m, 2H, CH_2_), 1.44–1.22 (m, 12H, CH_2_), 1.08 (d, 6H, *J* = 6.8 Hz, CH_3_), 0.88 (t, 3H, *J* = 6.6 Hz, 75Hz, CH_3_). ^13^C NMR (100 MHz, CDCl_3_) δ = 162.9, 162.8, 131.6, 131.4, 130.9, 130.8, 128.5, 127.0, 126.7, 116.7, 44.8, 41.1, 31.8, 29.3, 29.2, 28.1, 27.5, 27.1, 22.3, 20.1, 14.1. MS (FAB/NOBA (M + H)^+^) *m*/*z* 804.6. ν_max_/cm^−1^ 3062*w*, 2925*m,* 2855*w*, 1707*s*, 1660*s*, 1594*m*, 1558*s*, 1450*s*, 1340*m*, 1222*m*, 1170*m*, 1124*m*, 1002*m*, 820*m*, 767*s*, 717*m.* Anal. Calc. for C_44_H_39_F_3_N_6_O_6_: C 65.66%, H 4.88%, N 10.44%. Found: C 65.16%, H 5.01%, N 9.99%.

## 4. Conclusions

In summary, we have described the synthesis of two flavin derivatives functionalized with an NDI unit. The UV-Vis and CV data indicate that the flavin and NDI redox centers behave independently in the non-conjugated molecule **2**, and are coupled in conjugated analog **1**. The fluorescence emission studies clearly show that the flavin unit dominates the fluorescence properties of these systems, with substantial intramolecular quenching observed for the conjugated system **1**. The ability of **1** and **2** to accept multiple electrons coupled with their broad UV-Vis absorption properties make them promising acceptor molecules for organic photovoltaic systems; a direction we are currently exploring [21].

## Figures and Tables

**Table 1:** Summary of major absorptions derived from UV-Vis spectroscopy of compounds **1**, **2**, **10**, **7** and **9**.

Compound	λ^1^ (nm)	λ^2^(nm)	λ^3^(nm)	λ^4^(nm)	λ^5^(nm)
**10**	360	380			
**7**			418	434	459
**1**	359	379	414	434	455
**9**			419	437	462
**2**	359	380	420	437	462

**Table 2:** Cyclic Voltammetry data of **1**, **2**, **10** and parent flavins.

Compounds	*E*^1^_½_ (V)	*E*^2^_½_ (V)	*E*^3^_½_ (V)
**10**	−1.07	−1.52	
**7**	−1.02		
**1**	−0.95	−0.99	−1.47
**9**	−1.12		
**2**	−1.07	−1.53	
