# Correction:Zainalabdeen, N., *et al*., Synthesis and Characterization of Naphthalenediimide-Functionalized Flavin Derivatives. *Int. J. Mol. Sci.* 2013, *14*, 7468–7479.

**DOI:** 10.3390/ijms15034255

**Published:** 2014-03-10

**Authors:** Nada Zainalabdeen, Brian Fitzpatrick, Mohanad Mousa Kareem, Vikas Nandwana, Graeme Cooke, Vincent M. Rotello

**Affiliations:** 1 Glasgow Centre for Physical Organic Chemistry, WestCHEM, School of Chemistry, University of Glasgow, Glasgow G12 8QQ, UK; E-Mails: n_y92@yahoo.com (N.Z.); brianf@chem.gla.ac.uk (B.F.); mohanad_1972@yahoo.com (M.M.K.); 2 Department of Chemistry, University of Massachusetts, Amherst, MA 01003, USA; E-Mail: nandwana@chem.umass.edu

In the original version of the manuscript [1] some of the analytical data for compounds **1** and **2** were incorrect. The correct NMR data are presented below. The authors apologize for any inconvenience this may have caused to the readers of this journal.

Compound **1**:

^1^H NMR (500 MHz, DMSO-d6) δ 11.64 (s, 1H), 8.73 (s, 4H), 8.57 (d, *J* = 1.4 Hz, 1H), 8.16 (dd, *J* = 8.9, 1.4 Hz, 1H), 7.81 (d, *J* = 8.5 Hz, 2H), 7.64 (d, *J* = 8.5 Hz, 2H), 6.99 (d, *J* = 8.9 Hz, 1H), 4.08 (t, *J* = 7.0 Hz, 2H), 3.28 (m, 2H), 1.69 (quin, *J* = 7.0 Hz, 2H), 1.33 (m, 8H), 0.86 (t, *J* = 6.8 Hz, 3H). ^13^C NMR (125 MHz, DMSO-d6) δ 162.6 (2xC = 0), 162.3 (2xC = 0), 158.9, 155.1, 151.9, 140.8, 136.6, 136.1, 135.2, 133.7, 131.1 (2xC), 130.5 (4xC), 130.3 (q, *J* = 4 Hz), 128.6 (q, *J* = 4 Hz), 128.4 (2xC), 126.6, 126.5 (2xC), 126.4 (q, *J* = 31 Hz), 126.3 (2xC), 126.2, 123.2 (q, *J* = 271 Hz), 117.8, 39.9, 30.9, 28.5, 28.3, 27.1, 26.3, 21.9, 13.7.

Compound **2**:

^1^H NMR (500 MHz, CDCl_3_) δ 8.77 (s, 4H), 8.58 (d, *J* = 1.4 Hz, 1H), 8.03 (dd, *J* = 9.1, 1.4 Hz, 1H), 7.87 (d, *J* = 8.4 Hz, 2H), 7.76 (d, *J* = 9.1 Hz, 1H), 7.27 (d, *J* = 8.4 Hz, 2H), 5.37 (s, 2H), 4.61 (br s, 2H), 4.19 (t, 2H), 2.47 (sept, *J* = 6.7 Hz, 1H), 1.74 (m, 2H), 1.47–1.23 (m, 10H), 1.07 (d, *J* = 6.7 Hz, 6H), 0.87 (t, *J* = 6.9 Hz, 3H). ^13^C NMR (125 MHz, CDCl_3_) δ 163.1 (2xC = O), 162.9 (2xC = O), 159.0, 155.0, 149.9, 138.9, 137.5, 135.2, 134.9, 134.3, 131.7 (2xC), 131.5 (2xC), 131.2 (q, *J* = 4 Hz), 131.1 (4xC), 130.9 (q, *J* = 4 Hz), 128.6 (2xC), 127.1 (2xC), 127.0 (q, *J* = 28 Hz), 126.8 (2xC), 123.1 (q, *J* = 270 Hz), 116.9, 51.5, 44.9, 41.2, 31.9, 29.4, 29.3, 28.2, 27.6, 27.2, 22.8, 20.2 (2xC), 14.2.

The corrected version of the paper can be accessed at http://www.mdpi.com/1422-0067/15/3/4255/s1.
